# Antiviral Activity of Naringenin Against Equine Alphaherpesvirus Type 8 In Vitro

**DOI:** 10.3390/vetsci13101022

**Published:** 2026-09-27

**Authors:** Anrong You, Rongze Yang, Wei Wang, Enxin Li, Muhammad Zahoor Khan, Liangliang Li, Changfa Wang, Tongtong Wang, Juan Zhao

**Affiliations:** 1 College of Veterinary Medicine, Shanxi Agricultural University, Jinzhong 030600, China; 2 College of Agriculture and Biology, Liaocheng University, Liaocheng 252000, China

**Keywords:** naringenin, anti-EHV-8 infection, IFN response, HO-1

## Abstract

Equine alphaherpesvirus type 8 (EHV-8) is an economically significant emerging pathogen responsible for severe respiratory disease, reproductive failure, and neurological disorders in equids worldwide. The escalating prevalence of EHV-8 in large-scale donkey farming operations, particularly in China, has resulted in substantial economic losses; however, no licensed antiviral agents or prophylactic vaccines are currently available. In the present study, we evaluated the anti-EHV-8 activity of naringenin in susceptible cells and comprehensively explored the underlying molecular mechanisms. Our results demonstrated that naringenin markedly inhibited EHV-8 infection in RK-13 and NBL-6 cells in a dose-dependent manner. Moreover, naringenin blocked the replication of EHV-8 via HO-1 (heme oxygenase-1)-mediated type I interferon (IFN) response. Collectively, our findings indicate that naringenin may serve as a promising therapeutic drug for the treatment of EHV-8 infection.

## Simple Summary

Equine alphaherpesvirus type 8 (EHV-8) causes severe respiratory, reproductive, and neurological diseases in equids, with major economic losses in China, and no drugs or vaccines are available. We tested naringenin and found it inhibits EHV-8 infection in cells in a dose-dependent manner. Mechanistically, naringenin restores interferon production—which the virus suppresses—by HO-1 (heme oxygenase-1)-mediated type I interferon (IFN) response. Thus, naringenin is a promising therapeutic candidate, and our study provides new insights into flavonoid-mediated antiviral immunity.

## 1. Introduction

Equid alphaherpesvirus type 8 (EHV-8), also known as asinine herpesvirus type 3 (AHV-3), is a member of the alphaherpesvirinae subfamily that causes abortion, severe respiratory disease, and neurological disorders in horses and donkeys [1,2]. Phylogenetically, EHV-8 is closely related to equid alphaherpesvirus type 1 (EHV-1) and equid alphaherpesvirus type 4 (EHV-4), which are well-characterized pathogens causing substantial morbidity in equine populations globally [3]. In recent years, the prevalence of EHV-8 has been gradually increasing in large-scale donkey farms in China, which has caused severe economic losses in the donkey industry. EHV-8 infection in donkeys manifests as acute febrile illness, respiratory distress, abortion in pregnant mares, and, in severe cases, fatal encephalomyelitis [2,4,5]. However, limited knowledge impedes the development of effective vaccines or drugs to control EHV-8 infection.

Naringenin, a natural flavanone abundantly found in citrus fruits such as grapefruit, exhibits a diverse range of pharmacological activities, including anti-inflammatory, antioxidant, anticancer, anti-fibrotic, and immunomodulatory effects [6,7,8,9,10]. Crucially, naringenin demonstrates favorable biosafety and oral bioavailability profiles, making it an attractive scaffold for antiviral drug development. Notably, the potential antiviral activity of naringenin has emerged as a significant focus of research in recent years [11]. The antiviral effects of naringenin against several viruses, including porcine epidemic diarrhea virus (PEDV), pseudorabies virus (PRV), hepatitis C virus (HCV), and dengue virus serotype 2 (DENV2), have been investigated. For example, Xu et al. found that naringenin exerts anti-PRV activity in PK-15 cells by decreasing pro-inflammatory mediators’ expression via TLR4/NF-κB pathway activation [12]. Gong et al. reported that naringenin could decrease porcine epidemic diarrhea virus (PEDV) infection by interacting with the virus 3CLpro or PLP2 proteins [13]. Another study by Jia et al. showed that naringenin improves HCV infection-induced insulin resistance by regulating PTEN expression [14]. Lim et al. reported that naringenin suppresses DENV2 infection in vitro by targeting the DENV2 envelope protein [15]. Collectively, these studies establish naringenin as a versatile antiviral agent with activity against diverse RNA and DNA viruses, encompassing both enveloped and non-enveloped species. However, the anti-EHV-8 activity and potential mechanisms of naringenin are still unknown.

One practical caveat deserves explicit acknowledgement at the outset. Bitterness is not an incidental property of flavonoids but an evolved warning signal: bitter taste receptors (T2R/TAS2R) are widely held to have arisen so that animals can detect and reject plant secondary metabolites, and herbivores in particular rely on this modality to avoid potentially toxic forage [16]. Naringenin and its glycoside naringin are the principal bitter flavanones of citrus and are directly responsible for the bitterness of grapefruit [17]. Equids are no exception—horses discriminate bitter from sweet stimuli and answer bitter oral infusions with a stereotyped aversive gape and tongue protrusion [18]—and equine feed manufacturers routinely incorporate palatants expressly to mask bitterness and overcome feed neophobia [19]. For a compound intended for in-feed or in-water administration to donkeys, an unmitigated bitter profile therefore risks feed refusal and erratic dosing, which is precisely the failure mode that has limited the field application of many otherwise promising phytochemicals. Several complementary strategies could mitigate this liability. Molecular encapsulation is the most direct: complexation of naringenin with hydroxypropyl-β-cyclodextrin raises its aqueous solubility more than 400-fold and its plasma AUC and Cmax by 7.4- and 14.6-fold, respectively, in rats [17], while self-nanoemulsifying, liposomal and polymeric nanoparticle systems similarly sequester the aglycone from lingual TAS2Rs while correcting its poor oral bioavailability [20]. Co-formulation with species-appropriate sweeteners is also tractable, since the equine sweet receptor T1R2–T1R3 is activated by sucralose, steviol glycosides and neohesperidin dihydrochalcone—the last itself a citrus-derived bitterness-masking agent [21]—and apple- or caramel-type palatants measurably increase concentrate intake in ponies [19]. Selective TAS2R antagonists, such as the 6-methoxyflavanone derivatives that block hTAS2R39, offer a receptor-level rather than formulation-level solution [22]. Alternatively, because EHV-8 is principally a respiratory pathogen, nebulized or intranasal delivery would deposit the compound at the primary site of replication while bypassing the oral cavity altogether. Reassuringly, the practical scale of the problem may be modest: citrus flavanones have been supplied to livestock at gram-per-day doses without depressing dry matter intake [23]. We therefore regard bitterness as a solvable formulation problem rather than a disqualifying property of the naringenin scaffold.

The mitogen-activated protein kinase (MAPK) family—comprising the extracellular signal-regulated kinase (ERK1/2), c-Jun N-terminal kinase (JNK), and p38 branches—constitutes a set of evolutionarily conserved stress-sensing modules that convert extracellular and oxidative cues into transcriptional programs and that are extensively manipulated by herpesviruses during infection [24,25]. One major downstream effector of these cascades is the nuclear factor erythroid 2-related factor 2 (Nrf2)/heme oxygenase-1 (HO-1) axis. Under homeostatic conditions, Nrf2 is sequestered in the cytoplasm by Kelch-like ECH-associated protein 1 (Keap1) and continuously targeted for proteasomal degradation; electrophilic or oxidative stimuli, together with MAPK-dependent phosphorylation of Nrf2, disrupt the Keap1–Nrf2 interaction and permit Nrf2 nuclear translocation and transactivation of antioxidant response element (ARE)-driven genes, of which HMOX1 (encoding HO-1) is the most prominent [26]. Beyond heme catabolism, HO-1 acts as a restriction factor for a range of viruses and reinforces type I IFN signaling. This axis is directly relevant to EHV-8: pharmacological induction of HO-1 with cobalt protoporphyrin (CoPP) restores IFN-α/β production and blocks EHV-8 replication [27], and HO-1 itself behaves as an EHV-8 restriction protein [28]. Critically, however, the three MAPK branches are not functionally interchangeable upstream of Nrf2, and the branch that a given phytochemical recruits determines the kinetics and magnitude of HO-1 induction [29,30]. However, identifying which MAPK branch is key question for naringenin against EHV-8.

The aim of the present study was to assess the antiviral activity of naringenin against EHV-8 and to elucidate its underlying mechanisms. Our research demonstrated that naringenin obviously suppresses EHV-8 replication in both RK-13 and NBL-6 cells, which disrupts EHV-8 infection at the post-entry phases. Furthermore, naringenin exerts anti-EHV-8 activity via promoting type I interferon generation mediated by HO-1. These findings suggest that naringenin holds promise as a potential therapeutic agent for EHV-8 control and therapy.

## 2. Materials and Methods

### 2.1. Cell and Viruses

Rabbit kidney cells (RK-13, CCTCC, China) and equine dermal cells (NBL-6, ATCC, USA) were cultured in Modified Eagle Medium (MEM) with 10% fetal bovine serum (FBS) supplemented with penicillin–streptomycin. The cultures were incubated at 37 °C in a humidified atmosphere containing 5% CO_2_. Three EHV-8 isolates were used in the present study: SDLC66 (GenBank: MW816102.1), SD2020113 (GenBank: MW822570.1), and donkey/Shandong/10/2021 (GenBank: OL856098.1). All EHV-8 strains were propagated in RK-13 cells, and viral titers were determined using the Reed–Muench method as previously described [4].

### 2.2. Cell Viability Detection Assay

Naringenin was obtained from Sparkiade Biotechnology Co., Ltd. (Jinan, China) and dissolved in dimethyl sulfoxide (DMSO, Solarbio, Beijing, China) to a concentration of 25 mM. The cytotoxicity of naringenin or MAPK inhibitors (SP600125, PD98059, and SB203580) in RK-13 or NBL-6 cells was determined using a Cell Counting Kit-8 (CCK-8, MCE, South Brunswick Township, NJ, USA) as previously described [29]. Briefly, RK-13 or NBL-6 cells were seeded into 96-well plates overnight and incubated with naringenin or MAPK inhibitors at various concentrations for 24 h. Subsequently, 10 µL of CCK-8 reagent was added to each well and incubated at 37 °C for 2 h. The absorbance at 450 nm was detected to determine the cell survival rate using an Epoch™ microplate spectrophotometer (BioTek, Winooski, VT, USA). DMSO-treated cells served as the negative control, and the 50% cytotoxic concentration (CC50) was analyzed using GraphPad Prism 8.0 software.

### 2.3. EHV-8 Infectivity Inhibition Assay

To explore the potential antiviral effects of naringenin on EHV-8 infection in vitro, RK-13 or NBL-6 cells were pre-treated with naringenin at 25, 50, or 100 µM for 2 h. DMSO-treated cells served as the negative control. Subsequently, these cells were infected with EHV-8 SDLC66 at a multiplicity of infection (MOI) of 0.1 for 1 h, after which the medium was replaced with 3% FBS MEM containing naringenin at the indicated concentrations. Finally, cell samples and supernatants were collected at 24 h post-infection (hpi) to detect EHV-8 replication using Western blot and TCID_50_ techniques.

### 2.4. RNA/DNA Extraction and qPCR Analysis

Cell samples were collected at the indicated time points, and total RNA was extracted using Trizol reagent (Invitrogen, Carlsbad, CA, USA). Subsequently, 1 µg of RNA from each group was converted into cDNA using the PrimeScript II First Strand cDNA synthesis kit (Takara, Kusatsu, Japan). EHV-8 gD gene expression was evaluated using specific primers (Table 1) via real-time PCR on a QuantStudio™ 1 Plus system (ABI, Waltham, MA, USA). GAPDH mRNA expression was used as an internal reference. Quantification of target gene expression was carried out using the 2^−ΔΔCt^ method [31].

### 2.5. Western Blot Detection

The Western blot assay was performed as previously described [32]. Briefly, cell samples were collected at the indicated time points and lysed using NP-40 lysis buffer (Solarbio, Beijing, China). The protein concentration was determined using a BCA protein assay kit (Thermo Scientific, Waltham, MA, USA). Equal amounts of protein were mixed with 5× loading buffer and separated on 12% SDS-PAGE gels. Subsequently, proteins were transferred onto a polyvinylidene difluoride (PVDF) membrane (Millipore, Billerica, MA, USA). After blocking the membrane with 5% skim milk, the PVDF membrane was incubated with primary antibodies (anti-gD antibody, anti-Nrf2, anti-HO-1 antibody, and anti-β-actin) overnight at 4 °C. After washing with PBS’T, the membrane was incubated with the appropriate HRP-conjugated secondary antibody. Protein bands were then visualized and imaged using a ChemiDoc MP imaging system (Bio-Rad, Hercules, CA, USA).

### 2.6. Indirect Immunofluorescence Assay

The antiviral activity against various EHV-8 strains was evaluated using an immunofluorescence assay (IFA) as previously described [33]. RK-13 or NBL-6 cells were cultured on glass slides in 12-well culture plates and pre-treated with naringenin (100 µM) for 2 h. Subsequently, these cells were infected with EHV-8 SD2020113 or donkey/Shandong/10/2021 at an MOI of 0.1. After infection, the medium was replaced with 3% FBS MEM containing 100 µM naringenin. These cells were fixed with paraformaldehyde at 36 hpi and permeabilized with 0.25% Triton X-100. After washing with PBS, the cells were incubated with mouse anti-EHV-8 serum (prepared in our laboratory) as the primary antibody, followed by incubation with DyLight 594-conjugated goat anti-mouse IgG as the secondary antibody. Finally, the coverslips were mounted onto slides using Prolong^®^ Gold antifade reagent containing 4′,6-diamidino-2-phenylindole (DAPI) (Thermo Fisher, Waltham, MA, USA), and fluorescence images were captured using a DMi8 microscope system (Leica, Wetzlar, Germany).

### 2.7. Analysis of EHV-8 Replication Cycle

To identify the specific stage of the EHV-8 life cycle affected by naringenin, NBL-6 cells were seeded in 12-well plates and exposed to naringenin (100 µM) and EHV-8 SDLC66 (0.1 MOI) under various experimental conditions, including all-stage treatment, pre-treatment, co-treatment, and post-treatment. Following incubation, cells were washed with PBS after treatment and harvested at 24 hpi to analyze viral replication using TCID_50_ and Western blot.

### 2.8. siRNA Knockdown Assay

To evaluate the role of Nrf2 and HO-1 in the anti-EHV-8 effects of naringenin, NBL-6 cells were seeded into 12-well plates and transfected with siRNA targeting Nrf2 (siNrf2), siRNA targeting HO-1 (siHO-1), or siRNA negative control (siNC) for 10 h. Subsequently, these cells were treated with 100 µM naringenin for 2 h and infected with EHV-8 SDLC66 (0.1 MOI). At 24 hpi, all cells were harvested to assess EHV-8 replication by Western blot. The siRNA sequences are listed in Table 2.

### 2.9. Statistical Analysis

All cell-based experiments were performed in triplicate. Data were analyzed using GraphPad Prism 8.0 (San Diego, CA, USA). Differences among groups were analyzed by one-way analysis of variance followed by the Bonferroni *post hoc* test or unpaired *t* tests. Statistical significance was indicated as follows: * *p* < 0.05, ** *p* < 0.01, *** *p* < 0.001.

## 3. Results

### 3.1. Naringenin Inhibits EHV-8 Infection in Susceptible Cells

To assess the inhibitory effects of naringenin against EHV-8, we first illustrated the chemical structure of naringenin (Figure 1A) and determined the cytotoxicity of naringenin in RK-13 and NBL-6 cells using the CCK-8 kit. As shown in Figure 1B, the maximum safe concentration of naringenin in these cells was 100 µM. However, CC_50_ could not be obtained, since no concentration induced a 50% reduction in cell viability within the tested range. Subsequently, RK-13 and NBL-6 cells were pre-incubated with various concentrations of naringenin for 2 h and infected with EHV-8 SDLC66 (MOI = 0.1). TCID_50_ and Western blot assays were performed to detect EHV-8 replication at 24 hpi. The results showed that naringenin decreased virus titer and gD protein levels in RK-13 cells in a dose-dependent manner (Figure 1C,D). Similar results were observed in NBL-6 cells (Figure 1E,F). These results demonstrated that naringenin inhibited EHV-8 infection in susceptible cell lines.

### 3.2. Antiviral Activity of Naringenin Against Different EHV-8 Strains

To further evaluate whether naringenin exerts antiviral effects against other EHV-8 strains, RK-13 or NBL-6 cells were pre-treated with 100 µM naringenin and then infected with EHV-8 strains SD2020113 or donkey/Shandong/10/2021. Cells were collected at 24 hpi to analyze viral replication by qPCR and Western blot. As demonstrated in Figure 2A,B, compared with the DMSO-treated control cells, the naringenin treatment obviously decreased gD expression at both the mRNA and protein levels in RK-13 cells. Similar reductions in gD expression at both the mRNA and protein levels were observed in NBL-6 cells (Figure 2D). Moreover, we performed an IFA assay using mouse anti-EHV-8 serum to confirm the anti-EHV-8 effects of naringenin. As shown in Figure 2E, naringenin significantly decreased the number of positive cells compared with DMSO-treated control cells (Figure 2F). These data indicated that naringenin exhibits broad-spectrum antiviral activity against different EHV-8 strains.

### 3.3. Naringenin Disrupts Post-Entry Stage of EHV-8 Infection

To determine whether naringenin can directly inactivate EHV-8 in vitro, EHV-8 SDLC66 (0.1 and 0.5 MOI) was mixed with naringenin (100 µM) or DMSO at 37 °C for 2 h then added to NBL-6 cells. Viral progeny yield and the gD expression of EHV-8 were assessed using TCID_50_ and Western blot analysis at 24 hpi. As shown in Figure 3A,B, no significant differences were observed in either viral progeny production or gD expression between the naringenin-treated and DMSO-treated groups. These findings suggest that naringenin does not exert a direct inactivating effect against EHV-8.

To further investigate the mechanism of naringenin-mediated EHV-8 inhibition, a time-of-addition analysis was performed in NBL-6 cells (Figure 3C). Four experimental groups were designed: All-stage treatment (naringenin treatment at all stages, including pre-treatment, co-treatment, and post-treatment); pre-treatment (naringenin pre-treatment only); co-treatment (naringenin and EHV-8 co-treatment); and post-treatment (naringenin post-treatment only). Cell supernatants and cells were harvested at 24 hpi to evaluate EHV-8 replication using TCID_50_ and Western blot. As shown in Figure 3D,E, compared with DMSO-treated control cells, viral progeny generation and gD expression were significantly lower in the all-stage treatment and post-treatment groups, indicating that naringenin inhibits the late stages of EHV-8 infection.

### 3.4. Naringenin Exerts Anti-EHV-8 Activity via Induction of IFN-α/β Generation

Previous studies found that naringenin inhibits PRRSV infection in Marc-145 cells by promoting type I interferon (IFN-I) production [34]. To test whether naringenin exerts its anti-EHV-8 activity via upregulation of IFN-α/β expression, NBL-6 cells were pre-treated with naringenin at various dosages and infected with or without EHV-8 SDLC66. qPCR was performed to analyze IFN-α/β expression at 24 hpi. As shown in Figure 4A,B, naringenin treatment enhanced IFN-α and IFN-β expression in a dose-dependent manner in NBL-6 cells.

### 3.5. Naringenin Exerts Anti-EHV-8 Effects via HO-1 Mediated IFN Production

Previous studies showed that the Nrf2/HO-1 signaling pathway plays a role in regulating IFN-α-mediated antiviral responses [29,35]. To test whether naringenin promotes IFN-α-related antiviral gene expression via Nrf2/HO-1 activation in NBL-6 cells, we evaluated Nrf2 and HO-1 expression in naringenin-treated NBL-6 cells at different concentrations using qPCR and Western blot. The results showed marked increases in both Nrf2 and HO-1 expression (Figure 5A,B). To further confirm the involvement of Nrf2/HO-1 in naringenin-mediated antiviral activity via induction of IFN-α/β generation, NBL-6 cells were pre-transfected with siNrf2 or siHO-1 and treated with naringenin (100 µM). As demonstrated in Figure 5C,D, knockdown of either Nrf2 or HO-1 expression significantly diminished IFN-α/β expression compared with siNC treatment, and gD expression was reversed with siNrf2 or siHO-1 treatment.

A previous reports found that p38, JNK, or ERK1/2 signal molecules of the mitogen-activated protein kinase (MAPK) signaling pathway play key roles in regulating cellular defenses against viral invasion [24,25,36,37,38]. To determine whether the MAPK signaling pathway participates in the anti-EHV-8 activity of naringenin, NBL-6 cells were incubated with 100 µM narinogenin for 2 h. The phosphorylation levels of p38, ERK1/2, and JNK were analyzed using Western blot. As shown in Figure 5E, naringenin treatment resulted in a gradual increase in p38 phosphorylation levels in a time-dependent manner. However, ERK1/2 and JNK phosphorylation showed no obvious changes at any time point. To further investigate the role of p38 in the HO-1-mediated induction induced by naringenin, NBL-6 cells were treated with a mixture of various MAPK inhibitors and naringenin, and HO-1 expression was evaluated. The results revealed that SB203580 (p38 inhibitor) treatment significantly decreased HO-1 expression induced by naringenin, compared with JNK inhibitor (SP600125) and ERK1/2 inhibitor (PD98059) treatment (Figure 5F). Furthermore, NBL-6 cells were incubated with a mixture of p38 inhibitor (SB203580) and naringenin and subsequently infected with EHV-8 SDLC66. The mRNA and protein levels of gD and HO-1 were analyzed using qPCR and Western blot. As expected, SB203580 treatment significantly reversed the anti-EHV-8 effects of naringenin, as evidenced by higher gD protein expression in this group compared with the naringenin-only treated group. These results demonstrate that the anti-EHV-8 effects of naringenin may be dependent on p38-mediated HO-1 induction (Figure 5G,H).

## 4. Discussion

In recent years, large-scale donkey farming has been increasing in China, where donkeys serve important roles in agriculture and traditional medicine production. Respiratory diseases and abortion have become serious bottlenecks restricting the development of the donkey breeding industry [39]. Our research group found that EHV-8 causes respiratory disease, abortion, and neurological disorders in donkeys, which has led to enormous economic losses for the donkey industry in China [40]. Currently, there are no approved vaccines or antiviral therapeutics for EHV-8 control, necessitating urgent investigation of potential control strategies. It is therefore urgent to develop new anti-EHV-8 drugs. In this study, we screened and identified the natural compound naringenin and investigated its anti-EHV-8 activity in vitro. Our results have demonstrated that naringenin exerts antiviral properties against EHV-8, establishing for the first time the anti-EHV-8 potential of this naturally occurring flavonoid.

Naringenin has been reported to possess various pharmacological properties, including antiviral, anti-inflammatory, antioxidant, anticancer, and anti-diabetic activities. Numerous studies show that naringenin exerts antiviral activity against several viruses, including SARS-CoV, PRV, HCV, CHIKV, DENV, and ZIKV [41]. Importantly, naringenin has also been shown to inhibit PRRSV infection in Marc-145 cells by reactivating the RIG-I-MAVS signaling pathway and promoting type I IFN production [34], suggesting that its antiviral mechanism involves the modulation of innate immune signaling rather than direct virucidal activity, a finding consistent with our current observations. However, the effect of naringenin on EHV-8 infection has not been explored. In the present study, our results indicated that naringenin inhibits infection by different EHV-8 strains in susceptible cells (Figure 1 and Figure 2), disrupting EHV-8 infection at the replication stage (Figure 3), which is consistent with a recent study reporting that naringenin inhibits PRV proliferation in PK-15 cells at the replication stage [12].

The type I IFN system, as a component of host innate immunity, plays a key role in restricting viral replication [42,43]. Herpesviruses, including EHV-8, have evolved multiple immune evasion strategies to counteract type I IFN responses, making the pharmacological restoration of IFN signaling a promising therapeutic approach.

The Nrf2/HO-1 axis is not a new target in EHV-8 biology, and the value of the present work lies in what it adds to, rather than repeats from, that literature. Five prior studies have converged on this axis (Table 3): cobalt protoporphyrin (CoPP), a synthetic metalloporphyrin HO-1 inducer, restores IFN-α/β and blocks EHV-8 replication in cells and mice [27]; HO-1 itself was subsequently shown to be an EHV-8 restriction protein, acting through its metabolite biliverdin via the PKCβ/ERK1/ERK2 and NO/cGMP/PKG pathways [28]; rutin, a quercetin-3-O-rutinoside, suppresses EHV-8 principally through Nrf2/HO-1-dependent relief of oxidative stress [44]; Celastrol, a triterpenoid quinone methide, induces IFN-α-related antiviral genes via Nrf2/HO-1 [33]; and our previous study on Hyperoside identified JNK as the kinase coupling that flavonol glycoside to Keap1/Nrf2/HO-1 [29]. The main difference is that Hyperoside signals through JNK [29], and HO-1-derived biliverdin acts downstream through ERK1/2 [28], whereas naringenin selectively increases p38 phosphorylation with no detectable change in JNK or ERK1/2 phosphorylation at any time point tested (Figure 5E); correspondingly, only the p38 inhibitor SB203580 abolishes naringenin-induced HO-1 expression and rescues viral gD (Figure 5F–H).

To further elucidate the underlying mechanisms of naringenin against EHV-8, our present results showed that the expression of type I interferon cytokines, which was suppressed by EHV-8 infection, was reversed by naringenin treatment (Figure 4). This finding is particularly significant, as it suggests that naringenin counteracts a key immune evasion mechanism of EHV-8, thereby restoring the host’s intrinsic antiviral defense capacity. The MAPK and Nrf2/HO-1 signaling pathways serve as pivotal regulators of intracellular defense against inflammatory responses and viral infection [26]. For instance, arctiin inhibits H9N2 virus infection by regulating inflammatory responses via the JNK MAPK/Nrf2/HO-1 signaling pathway [30]. Our present study revealed that naringenin promotes the expression levels of phosphorylated p38 (p-p38), Nrf2, and HO-1 in NBL-6 cells (Figure 5). Notably, these effects can be reversed by siNrf2, siHO-1, and the p38 inhibitor SB203580. Importantly, the selectivity of p38 in mediating naringenin’s effects—evidenced by the failure of the JNK and ERK1/2 inhibitors to attenuate HO-1 induction—defines a mechanistic axis distinct from that used by the flavonol glycosides hyperoside and rutin in the same virus–host system [29,44].

A limitation of the present study should be stated plainly. Unlike the CoPP, rutin and Celastrol studies [27,33,44], our analysis is confined to susceptible cell lines, and a murine model—followed by donkeys—remains necessary before naringenin can be advanced as a therapeutic candidate.

## 5. Conclusions

In summary, we demonstrated that naringenin can inhibit EHV-8 infection in susceptible cells via HO-1-mediated production of IFN-α/β (Figure 6), indicating that naringenin could be a potential novel drug for EHV-8 control in the future.

## Figures and Tables

**Figure 1:**
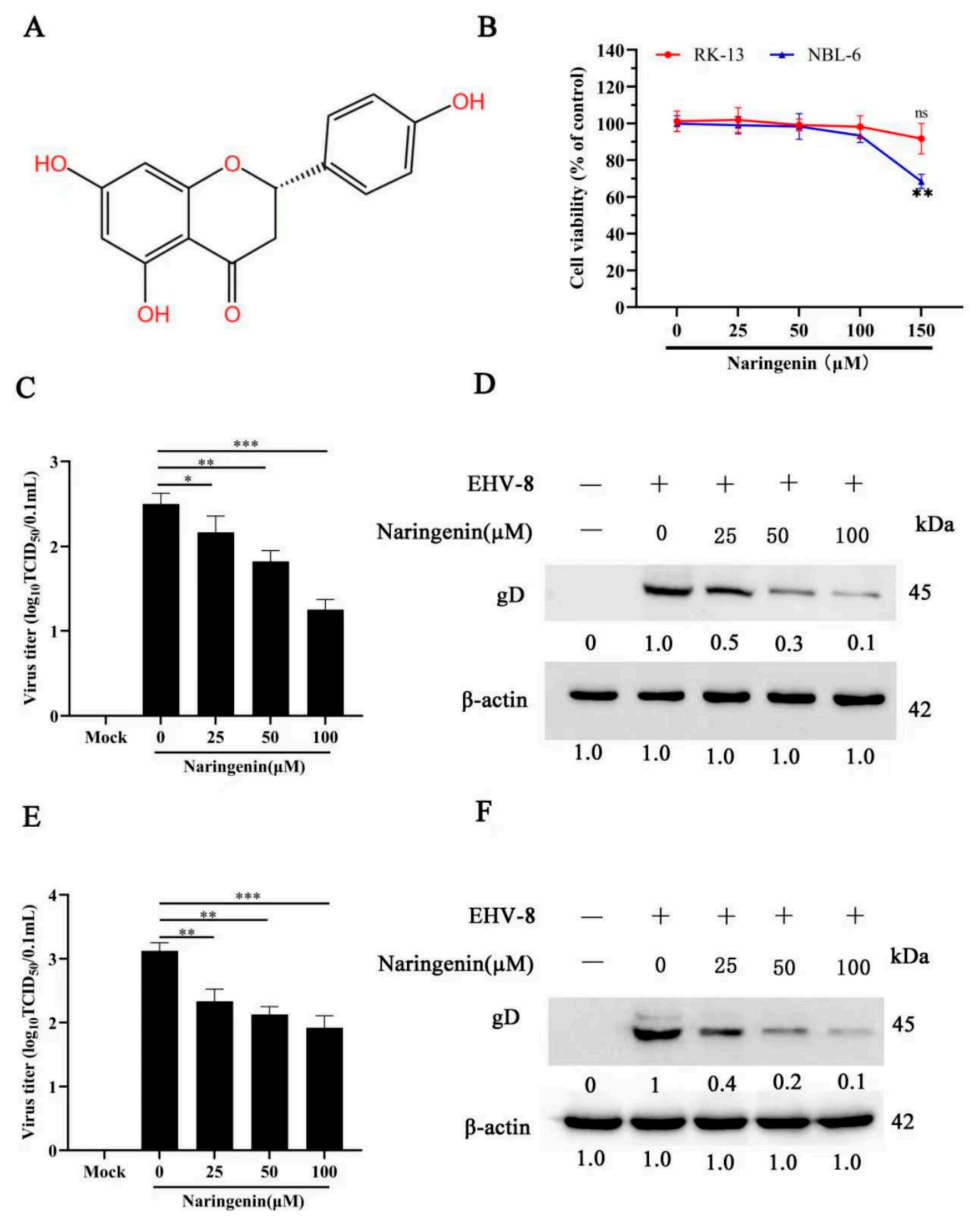
Identification of anti-EHV-8 activity of naringenin in susceptible cells. (**A**) The chemical structure of naringenin. (**B**) Cytotoxicity of naringenin in RK-13 and NBL-6 cells. Cells were incubated with naringenin at different concentrations (0, 25, 50, and 100 µM) for 24 h. The cell viability was measured using a CCK-8 kit. Results were confirmed in three independent experiments. ** *p* < 0.01 vs. the DMSO-treated group. RK-13 (**C**,**D**) and NBL-6 (**E**,**F**) cells were preincubated with naringenin at the indicated concentrations (25, 50, and 100 µM) for 2 h, followed by infection with EHV-8 SDLC66 at 0.1 MOI. Progeny virus production and gD protein expression were analyzed by TCID_50_ and Western blot at 24 hpi. β-Actin served as the internal loading control. Results represent data from three independent experimental replicates analyzed using one-way ANOVA followed by the Bonferroni *post hoc* test. The original images of the Western blots shown in panels (**D**,**F**) are provided in Figure S1. ns: no difference, * *p* < 0.05, ** *p* < 0.01, *** *p* < 0.001 vs. the DMSO-treated group (0 µM naringenin).

**Figure 2:**
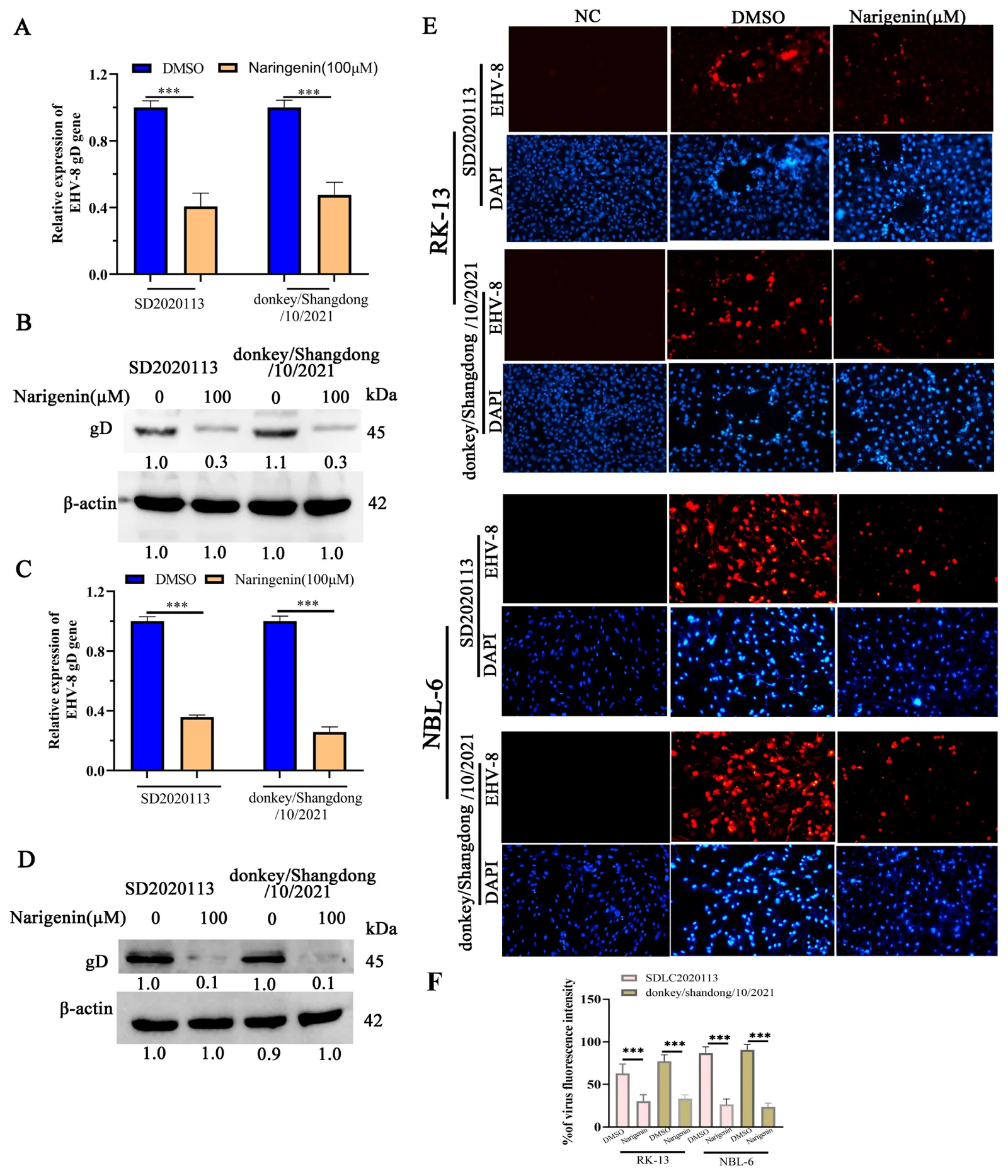
Naringenin exhibits a broad-spectrum anti-EHV-8 activity in vitro. RK-13 (**A**,**B**) and NBL-6 (**C**,**D**) cells were pre-treated with 100 µM naringenin or an equivalent volume of DMSO for 2 h, respectively, and then infected with EHV-8 SD2020113 or EHV-8 Donkey/Shandong/10/2021 at 0.1 MOI. Cell supernatants and cell lysates were harvested at 24 hpi to quantify viral load and gD expression by qPCR and Western blot, respectively. Data are presented as mean ± SD from at least three independent experiments. *** *p* < 0.001 vs. the DMSO-treated group. (**E**) RK-13 and NBL-6 cells were pre-treated with 100 µM naringenin or DMSO for 2 h and then infected with EHV-8 SD2020113 or EHV-8 Donkey/Shandong/10/2021 at 0.1 MOI. Cells were fixed with paraformaldehyde at 36 hpi for the immunofluorescence assay (IFA). Mouse anti-EHV-8 positive serum (red) and 4′,6-diamidino-2-phenylindole (DAPI; blue) were used to detect EHV-8 antigens and stain cell nuclei, respectively. Images were captured using a Leica DMi8 microscope, and histograms represent the percentage of EHV-8 virus fluorescence intensity, performed using ImageJ software 1.54 version and based on 5 regions/sample (**F**). The original images of the Western blots shown in panels (**B**,**D**) are provided in Figure S2. *** *p* < 0.001 vs. the DMSO-treated group. scale bars = 100 µm.

**Figure 3:**
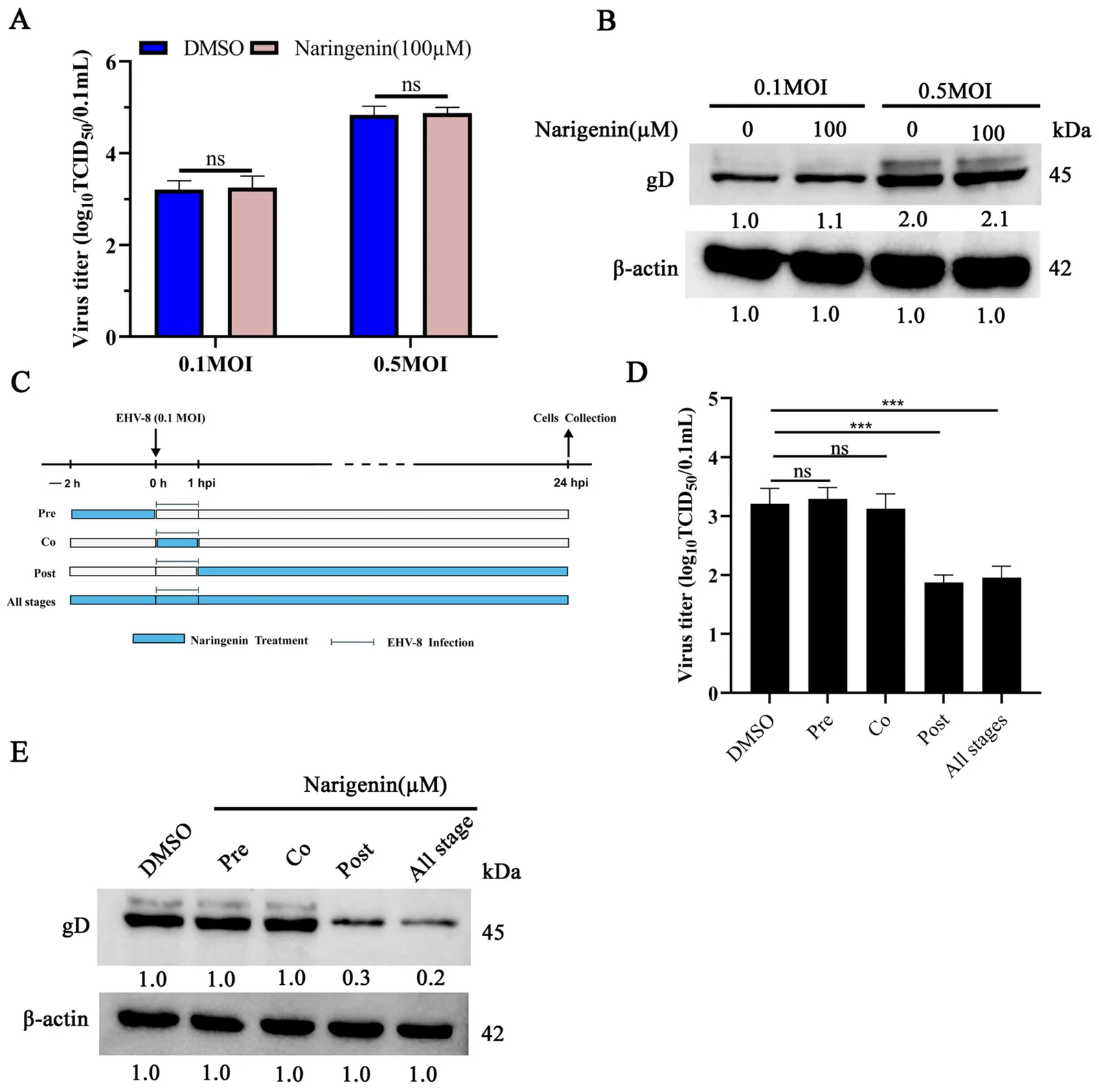
Naringenin disrupts EHV-8 replication at the late stages. EHV-8 SDLC66 (0.1 or 0.5 MOI) was premixed with 100 µM naringenin at 37 °C then incubated into NBL-6 cells. Cell supernatants and cell lysates were collected at 24 hpi to determine progeny virus production (**A**) and gD expression (**B**) by TCID_50_ and Western blot, respectively. (**C**) Schematic of the time-of-addition assay. NBL-6 cells were treated with 100 µM naringenin and infected with EHV-8 SDLC66 at 0.1 MOI at different time points, corresponding to all-stage treatment, pre-treatment, co-treatment, and post-treatment. The cell supernatants and cell lysates were collected to assess progeny virus production (**D**) and gD expression (**E**) by TCID_50_ and Western blot, respectively. Data are presented as mean ± SD from three independent experiments. The original images of the Western blots shown in panels (**B**,**E**) are provided in Figure S3. ns: no difference, *** *p* < 0.001 vs. the DMSO-treated group.

**Figure 4:**
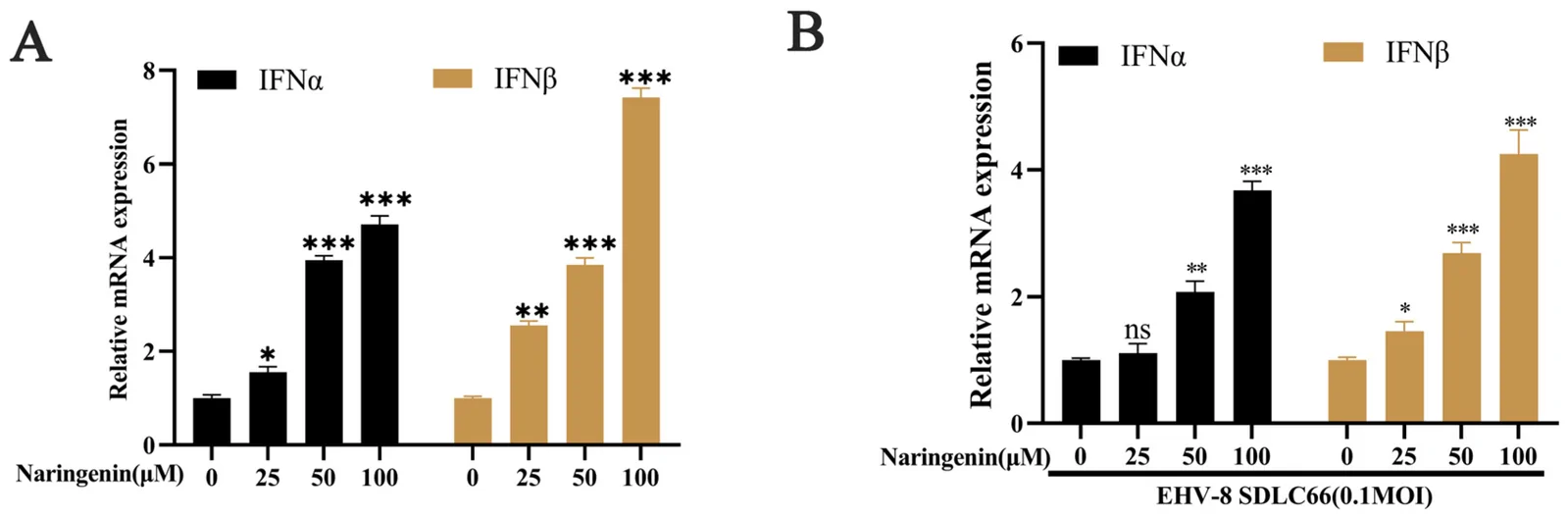
Naringenin exerts anti-EHV-8 activity by promoting type I IFN production. (**A**,**B**) NBL-6 cells seeded in 12-well plates were pre-treated with naringenin at different concentrations (0, 25, 50, and 100 µM) for 2 h, followed by infection with or without EHV-8 SDLC66 at 0.1 MOI. The mRNA levels of IFN-α and IFN-β were quantified by qPCR. GAPDH was used as the internal control. Results represent data from three independent experimental replicates analyzed using one-way ANOVA followed by Bonferroni *post hoc* test statistical analysis. * *p* < 0.05, ** *p* < 0.01, *** *p* < 0.001 vs. the 0 µM naringenin-treated group.

**Figure 5:**
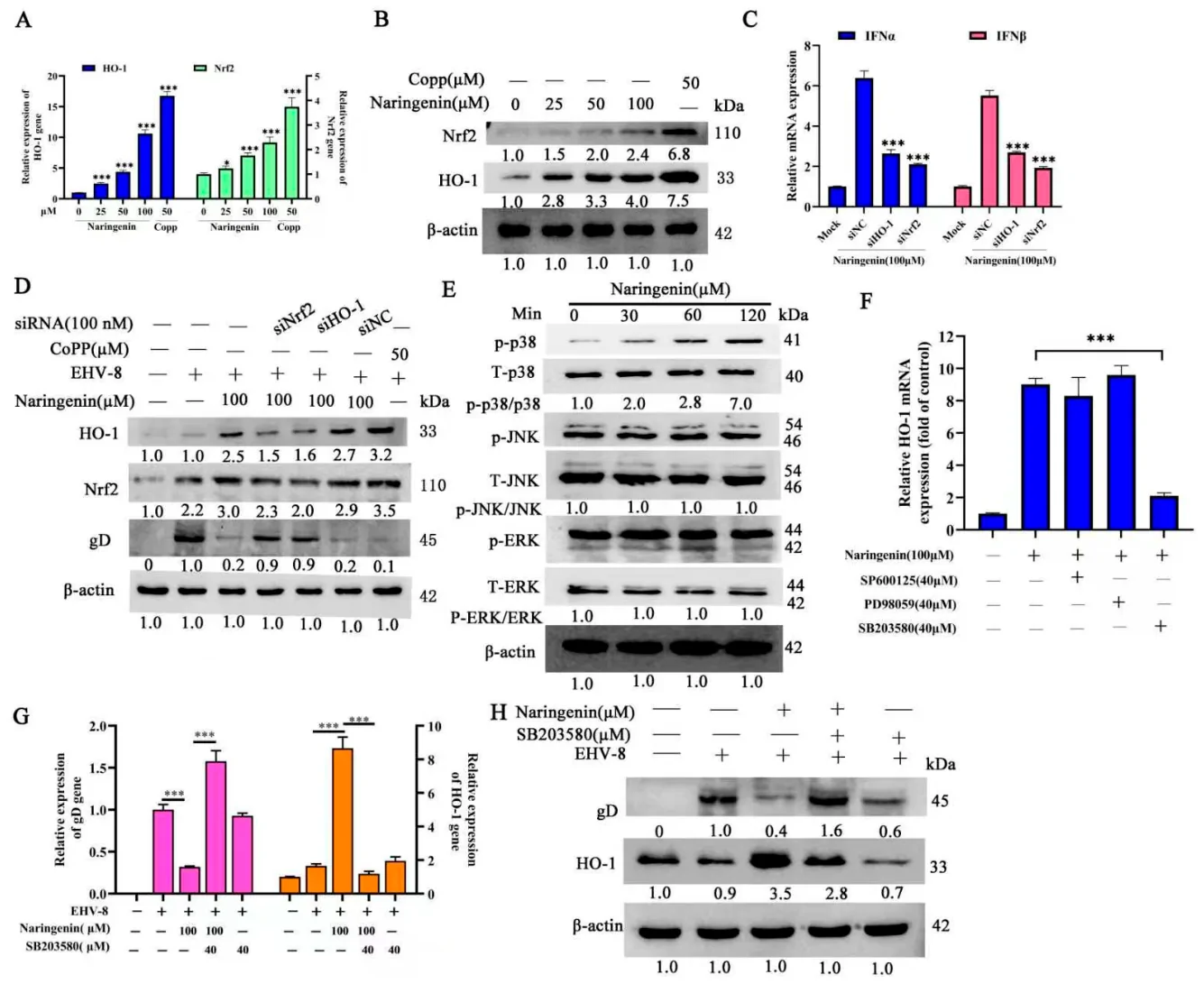
Naringenin exerts anti-EHV-8 activity via HO-1-mediated IFN production. NBL-6 cells were treated with naringenin (0, 25, 50, and 100 µM) for 24 h and then harvested to detect Nrf2 and HO-1 expression by qPCR (**A**) and Western blot (**B**). Cobalt protoporphyrin (CoPP) was used as the positive control. * *p* < 0.05; *** *p* < 0.001. (**C**) NBL-6 cells were pre-transfected with siRNA targeting Nrf2 (siNrf2), siRNA targeting HO-1 (siHO-1), or non-targeting control siRNA (siNC) for 10 h, followed by treatment with 100 µM naringenin for 2 h. Cells were harvested at 24 hpi to detect IFN-α and IFN-β expression by qPCR. GAPDH served as the internal control. *** *p* < 0.001. (**D**) NBL-6 cells were transfected with siNrf2, siHO-1, or siNC for 10 h and then treated with naringenin (100 µM) for 2 h prior to infection with EHV-8 SDLC66 (MOI of 0.1). The expression of Nrf2, HO-1, and viral gD protein was assessed by Western blot at 24 hpi. β-Actin served as the loading control. (**E**) Naringenin increased p38 phosphorylation. NBL-6 cells were treated with 100 µM naringenin and collected at the indicated time points (0, 30, 60, and 120 min) to analyze the phosphorylated and total levels of p38, JNK, and ERK1/2 by Western blot. (**F**) The p38 inhibitor SB203580 decreased HO-1 expression. NBL-6 cells were incubated with 100 µM naringenin combined with the ERK1/2 inhibitor (PD98059), the JNK inhibitor (SP600125), or the p38 inhibitor (SB203580) at 40 µM for 2 h; cells were then collected to assess HO-1 expression by qPCR. Data are presented as mean ± SD from three independent experiments. *** *p* < 0.001 vs. the naringenin-treated group. NBL-6 cells were incubated with a mixture of SB203580 (p38 inhibitor) and naringenin then infected with EHV-8 SDLC66 at 0.1 MOI. The mRNA and protein levels of gD and HO-1 at 24 hpi were analyzed using qPCR (**G**) and Western blot (**H**). The original images of the Western blots shown in panels (**B**,**D**,**E**,**H**) are provided in Figure S4.

**Figure 6:**
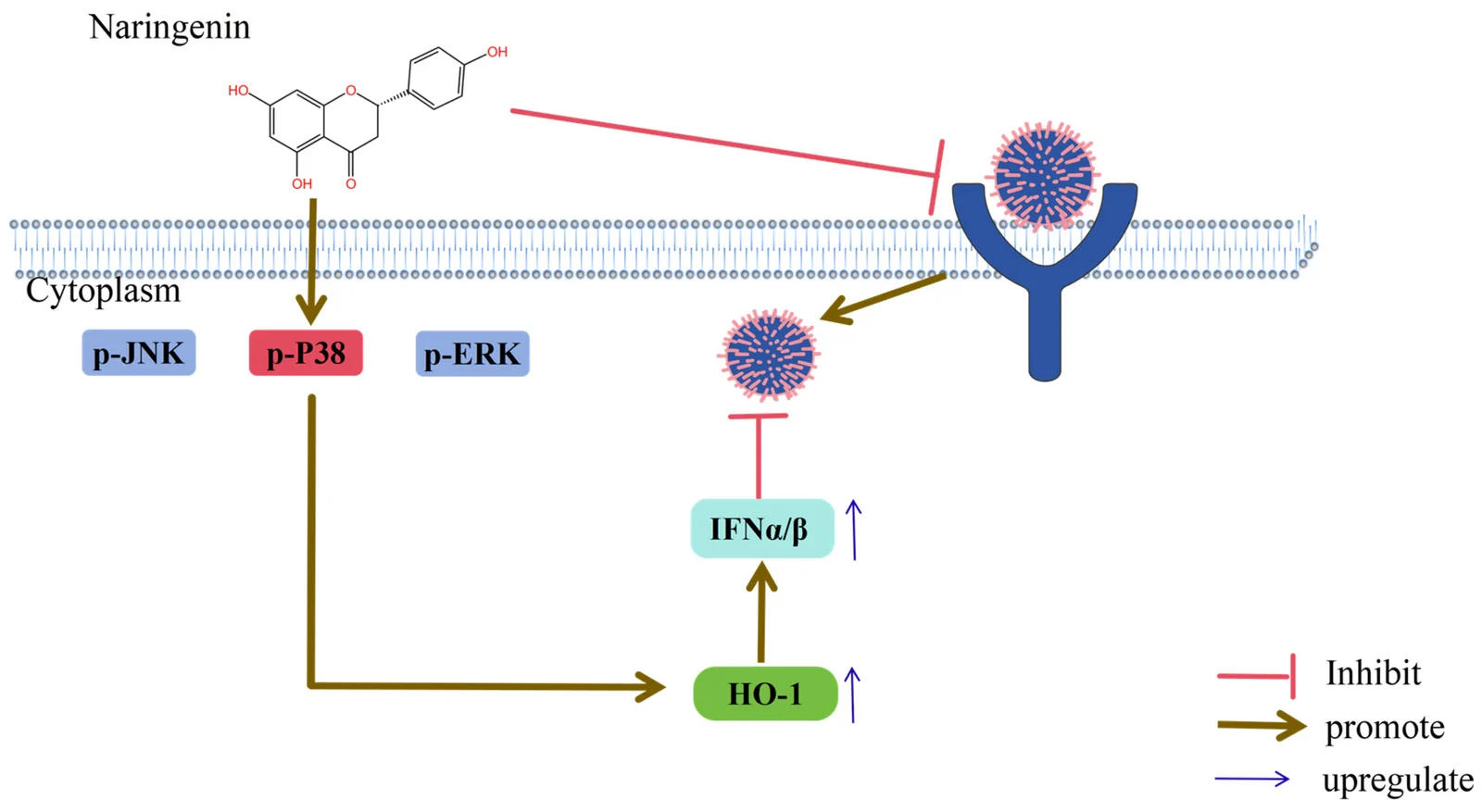
The scheme diagram of the naringenin against EHV-8 infection mechanism. Naringenin inhibits EHV-8 replication in susceptible cells via upregulating HO-1, thereby promoting the host type I IFN response.

**Table 1:** List of primers used in this study.

Genes	Forward Primer (5′-3′)	Reverse Primer (5′-3′)
*ORF72*	CCCACGTGTGCAACGCCTAT	ATACAGTCCCGAGGCAGAGT
*HO-1*	AGTTCATGAAGAACTTTCAGA	TACCAGAAGGCCATGTCC
*Nrf2*	ATTCAATGATTCTGACTCTG	CGTATCCCCAGAAGAATGTA
*IFNα*	TACTCAGCAGACCTTGAACCT	CAGTATTGGCAGCAAGTTGAC
*IFNβ*	AGCTCCAAGAAAGGACGA	GCCCTGTAGGTGAGGTTGATCT
*GAPDH*	GTCTCCTCTGACTTCAACAGCG	ACCACCCTGTTGCTGTAGCCAA
*β-actin*	ACGGCATCGTCACCAACTG	CAAACATGATCTGGGTCATCTTCT

**Table 2:** The sequence of siRNA used in this study.

Primers	Sequences (5′-3′)
siNrf2	CTCCTTAAGAAGCAACTCA
siHO-1	GGTCCTCACACTCAGCTTT
siNC	TTCTCCGAACGTGTCACGTTT

**Table 3:** Comparison of reported Nrf2/HO-1-directed anti-EHV-8 agents.

Agent	Chemical Class	Upstream Node	Effector Axis	Model	Ref.
CoPP	Metalloporphyrin	Not defined	HO-1 → IFN-α/β	Cells + mice	[27]
HO-1/biliverdin	Endogenous host protein/metabolite	PKCβ	ERK1/2; NO/cGMP/PKG	Cells + mice	[28]
Rutin	Flavonol glycoside (quercetin-3-O-rutinoside)	Not defined	Nrf2/HO-1 (antioxidant)	Cells + mice	[44]
Celastrol	Triterpenoid quinone methide	Not defined	Nrf2/HO-1 → IFN-α	Cells + mice	[33]
Hyperoside	Flavonol glycoside (quercetin-3-O-galactoside)	JNK	Keap1/Nrf2/HO-1 → IFN	Cells	[29]
Naringenin	Flavanone aglycone	p38 (JNK/ERK1/2 excluded)	HO-1 → IFN-α/β	Cells	This study

## Data Availability

The original data obtained in the study are included in the article. Further inquiries can be directed to the corresponding authors.

## Supplementary Materials

The following supporting information can be downloaded at: https://www.mdpi.com/article/10.3390/vetsci13101022/s1, Figure S1: Western blot images of gD and β-actin corresponding to Figure 1D,F; Figure S2: Western blot images of gD and β-actin corresponding to Figure 2B,D; Figure S3: Western blot images of gD and β-actin corresponding to Figure 3B,E; Figure S4: Western blot images of Nrf2, HO-1, gD, β-actin, p-ERK, p-JNK, p-p38, T-ERK, T-JNK, and T-p38 corresponding to Figure 5B,D,E,H.
